# Assessing the evidence for effective biosafety risk management in *Coxiella burnetii* research

**DOI:** 10.1016/j.onehlt.2025.101159

**Published:** 2025-08-05

**Authors:** S.D. Blacksell, K.K. Le, L.J. Gleeson, J. Stenos, S.R. Graves, N.P.J. Day

**Affiliations:** a Mahidol Oxford Tropical Medicine Research Unit, Faculty of Tropical Medicine, Mahidol University, Bangkok, Thailand; b Centre for Tropical Medicine and Global Health, Nuffield Department of Medicine, Nuffield Department of Medicine Research Building, University of Oxford, Oxford, United Kingdom; c Australian Rickettsial Reference Laboratory, University Hospital Geelong, Geelong, Victoria, Australia

**Keywords:** *Coxiella burnetii*, Q fever, Transmission, Biosafety evidence

## Abstract

*Coxiella burnetii*, the causative agent of Q fever, is a highly infectious zoonotic bacterium presenting significant biosafety challenges, particularly in laboratory environments. Due to its low infectious dose, environmental resilience, and potential for aerosol transmission, *C. burnetii* has been classified as a Risk Group 3 pathogen and Category B biothreat agent. This review evaluates the biosafety practices required for safe handling, including adherence to Biosafety Level 3 (BSL-3) containment for propagative work and BSL-2 standards for diagnostic activities. Strategies such as vaccination, personal protective equipment (PPE), environmental controls, and rigorous decontamination protocols are critical to mitigate laboratory-acquired infections (LAIs). Historical reports highlight *C. burnetii* as a leading cause of LAIs, underscoring the importance of implementing risk-based biosafety frameworks outlined by the World Health Organization. However, gaps remain in disinfection efficacy data, vaccine accessibility, and consistent application of biosafety measures, particularly in resource-limited settings. Addressing these challenges through enhanced surveillance, interdisciplinary research, and improved access to preventive tools can reduce *C. burnetii*-associated risks. This review emphasises the need for adaptive, sustainable practices to ensure global laboratory safety and highlights the integration of evidence-based strategies for *C. burnetii* research.

## 1 Introduction

Effective laboratory biosafety is critical for managing the risks associated with infectious pathogens, particularly in environments with limited resources. The World Health Organization (WHO) introduced the fourth edition of its Laboratory Biosafety Manual (LBM4) in 2020 [1], recognising the need for a more adaptive and sustainable approach to laboratory safety. This updated manual represents a shift from prescriptive biosafety measures to a risk-based approach, which emphasises tailoring safety practices to the specific hazards and operational contexts of individual laboratories. By focusing on assessing and managing risks, this approach enables laboratories to implement practical and scalable biosafety measures, even in resource-constrained settings [2]. The risk-based framework outlined in LBM4 prioritises flexibility and sustainability, encouraging laboratories to evaluate their unique risk profiles and adapt safety protocols accordingly. This paradigm shift is particularly relevant for handling pathogens such as *Coxiella burnetii*, the causative agent of Q fever in humans, which poses distinct biosafety challenges due to its environmental resilience, zoonotic nature, and the risks it presents for laboratory-acquired infections. The application of risk-based biosafety practices is crucial for laboratories working with *C. burnetii*, as these practices allow for more efficient allocation of resources while maintaining high standards of safety and containment. This review explores the risk-based biosafety strategies recommended by the WHO and their application to *C. burnetii*. It examines the unique characteristics of the pathogen, the current evidence base for laboratory safety practices, and the gaps in our understanding of effective risk management. By focusing on practical and adaptable measures, this review highlights how the risk-based approach can support safer laboratory practices for *C. burnetii*, particularly in settings with limited resources, while advancing the global commitment to sustainable biosafety practices.

## 2 General characteristics

*C. burnetii* is an obligate intracellular bacterium belonging to the order Legionellales and family Coxiellaceae, responsible for causing Q fever in humans and coxiellosis in animals. The bacterium is pleomorphic, ranging in size from 0.3 to 2.0 μm [3]. *C. burnetii* has two distinct morphologies: the Small Cell Variant (SCV), which possesses high virulence and is a “spore-like” form and the Large Cell Variant (LCV), which exhibits low transmissibility and is the replicating form [4]. The SCV infectivity can exhibit extended survival periods and heightened resistance to various environmental stressors [5]. Symptoms of Q fever vary, ranging from asymptomatic infection to acute or chronic illness. Acute Q fever typically presents with flu-like symptoms, including high fever, severe headache, muscle pain, fatigue, and pneumonia. Chronic Q fever is more severe but less common and can result in life-threatening complications such as endocarditis, particularly in individuals with pre-existing heart valve abnormalities or immunosuppression [6].

### 2.1 Reservoir and transmission

The transmission of *C. burnetii*, the causative agent of Q fever, involves a complex network of animal reservoirs and environmental factors. Mammalian species such as goats, cattle, sheep, and rats serve as primary hosts, while ticks also play a significant role in maintaining the bacterium's life cycle. Several tick species have been identified as *C. burnetii* carriers, including *Hyalomma aegyptium* [7], *Ixodes ricinus*, and *Dermacentor marginatus* [8], including ticks from domestic pets like dogs [9] [10]. Although ticks contribute to maintaining *C. burnetii* in the environment, the majority of human infections stem from aerosols and direct contact with infected animals or their products of parturition.

### 2.2 Treatment

In 2013, the Centers for Disease Control and Prevention (CDC) issued comprehensive guidance stating that routine antibiotic prophylaxis is not recommended following Q fever exposure. Instead, exposed individuals should monitor for symptoms (especially fever) for at least 3 weeks and begin doxycycline therapy promptly if symptoms develop within 6 weeks. Prophylaxis may be considered only in exceptional cases, such as high-risk intentional exposures (e.g., bioterrorism) [11].

### 2.3 Laboratory-acquired Q fever infections

Multiple studies have compiled cases of Q fever acquired in laboratory settings. Although there are some discrepancies among the studies, the significance of the problem and the risks posed by the pathogen are evident in the summary of reports presented in Table 1. Phillips [12] noted that between 1910 and 1950, 78 cases of Q fever were reported in the US Public Health Service laboratories In the same study, a survey of 65 laboratories in 18 countries in the late 1950s and early 1960s found that Q fever was the second most common laboratory-acquired infection (LAI), accounting for 96 cases [12]. In the United States, Sulkin and Pike reported that before 1951, 104 cases had occurred in laboratory settings [13], and 80 cases were reported between 1950 and 1963 [14]. Interestingly, a study cataloguing LAIs in peer-reviewed articles and government reports did not record any Q fever infections between 2001 and 2021 [15].

**Table 1:** Description of *Coxiella burnetii* laboratory-acquired infections.

Year	Location	Number of people affected	Description	
1940	NIH, Bethesda Maryland USA	15	“At least four significant laboratory outbreaks of Q fever have been reported in the literature since 1940. In that year, during a 54-day period, 15 of 153 persons working in one laboratory building of the old National Institutes of Health developed roentgenological, clinical, and serological evidence of Q fever pneumonitis. Work with Q fever rickettsia was confined to a laboratory on the second floor.”	[12,51]
1946	15th U.S. Medical General Laboratory, Italy	20	“In 1946 an outbreak of Q fever occurred among the personnel of the 15th U.S. Medical General Laboratory in Italy. As a result of the detection of the disease in American and British troops in that country, infected guinea pigs had been sent to the general laboratory for investigation. In a three-month period following receipt of the animals there were 20 cases of Q fever among the laboratory personnel or visitors to the laboratory. It was concluded that “the most likely mode of transmission was by inhalation of the infectious agent which became suspended in the air of the laboratory in spite of reasonable precautions.”	[12,52]
1946	Fort Bragg, North Carolina, USA	16	“In the same year (1946) at Fort Bragg, North Carolina, there were 16 cases of Q fever among personnel of the laboratories of the Commission on Acute Respiratory Diseases. Infected personnel worked in several different rooms of a laboratory building. The spread of the infecting agent, again by air-borne means, was felt to be connected with the processing and harvesting of infected eggs.”	[12,53]
1945–1946	NIH, Bethesda Maryland USA	47	“The largest reported laboratory outbreak of Q fever occurred in a single building of the National Institutes of Health between December 1945 and May 1946. A total of 47 persons, including persons who had merely visited the building for a short time, became infected. No infections occurred in persons working in the Q fever laboratory on the first floor. Almost all had either previously been infected or were immune.”	[12,54]

### 2.4 Non-laboratory occupational Q fever infections

Occupational exposure to infected animals remains a significant risk factor for *Coxiella burnetii* infection. Key incidents include a U.S. medical school outbreak linked to contact with pregnant ewes [16] and elevated infection rates among butchers and abattoir workers involved in camel slaughter in southeast Iran [17]. Livestock such as cattle, goats, and sheep are recognised as primary reservoirs for the bacterium, with infection rates of 46.6 %, 21.4 %, and 9.4 %, respectively [18]. Beyond animal reservoirs, *C. burnetii* demonstrates exceptional environmental persistence, persisting in settings with high human activity, such as farms, schools, and retail stores. Even contaminated clothing has been identified as a potential transmission vector [19,20]. Environmental contamination has also resulted in large-scale outbreaks, such as in central Israel, where over 300 individuals were infected through contaminated aerosols spread by air conditioning systems [21]. Another outbreak in Canada was attributed to the inhalation of textile dust [22]. Environmental conditions significantly influence the bacterium's airborne spread. For instance, strong November winds in southern France were linked to a surge in Q fever cases due to bacterial dispersion [23]. In contrast, areas with dense vegetation and high humidity tend to inhibit airborne transmission [24]. High social vulnerability and little-investigated settings from Latin America reveal important risks, with one study reporting a 33.7–41.0 % *C. burnetii* seroprevalence and a 14.5 % one-year seroincidence among incarcerated women in Brazil [25], and another showing an 8.1 % seroprevalence among wildlife professionals in Paraná State [26]—suggesting occupational and environmental exposure risks even in the absence of identified statistical associations.

## 3 Biosafety considerations and existing biosafety measures

Biosafety evidence published in peer-reviewed articles for *C. burnetii* and Q fever is summarised below and in Table 2.

**Table 2:** Detailed pathogen biosafety evidence for *Coxiella burnetii.*

Hazards	Hazard identification	Evidence (direct quote)	Reference
Modes of Transmission	Aerosols	“The air conditioning ducts were located on the dining room's roof and could be accessed by animal secretions. One of the 4 filter samples, as well as 1 of the 8 gauze swabs taken from the inlet of the dining room's air-conditioning unit, had positive results for Q fever by PCR. Similar positive PCR results were obtained by the Centers for Disease Control and Prevention on filter samples.”	[50]
		“Exposure to infected sheep varied greatly, from handling a placenta to a casual walk past a sheep cart in a corridor…One hundred thirty (39.4 %) of the 330 persons with possible exposure and two (4.3 %) of the 46 with no known exposure were seropositive…The 109 first-year medical students were based on the fourth floor, but 99 had seen sheep on the third floor at some time. Seven of these 99 students were seropositive.”	[16]
		“…representing 93.5 % of the wind in November to December 1998 but only 63.1 %, 47.7 %, and 85 % in November to December 1996, 1997, and 1999, respectively. All these differences were statistically significant, except those between 1998 and 1999 in the speed range > 5 m/s.”	[23]
		“Areas without transmission had a higher vegetation density and relatively shallow groundwater conditions. Vegetation and soil moisture are relevant factors in the transmission of *Coxiella burnetii* from infected farms to humans, by reducing the amount of dust available for dispersion of the bacteria.”	[24]

	Direct contact	“Serum from the cat had high antibody titres to phase I and phase II *Coxiella burnetii* antigens. The attack rate among the employees where the cat owner worked, 13 of 19 (68 %), was higher than that of employees elsewhere, 3 of 13 (28 %) [P less than 0.01]. The cat owner's wife and son also developed Q fever. None of the family members of the other employees with Q fever was so affected.”	[22]
		“In our study, the seroprevalence rate of phase I and II antibodies in butchers and slaughterhouse workers of Sistan va Baluchestan were 18.1 % and 14.4 %, respectively.”	[17]
		“At each site, an effort was made to represent areas of primarily human activity (post offices, grocery stores, etc.) in about 50 % of the locations and to represent areas with expected animal activity (dairy farms, ranches, etc.) in the remainder…Approximately 270 samples were taken in each state, with a total of 1622 samples included in the analysis. Out of these 1622 samples, 386 were positive by a PCR assay that specifically detects the IS1111 insertion sequence of *C. burnetii*”	[19]
		“Semen samples were donated by the index patient at 4, 15, and 23 months after the onset of the acute illness. The first 2 samples were readily found to be positive for *C. burnetii* DNA by PCR assay…”	[55]
		“…a patient who developed an acute febrile illness 85 days after receiving an allogeneic bone marrow transplant for acute myeloid leukaemia. Serological investigation indicated the cause to be Q fever.”	[56]

Infectious dose		“The one-hit model combined with our N estimate would mean that previous World Health Organization (1970) estimates of casualties resulting from use of *C. burnetii* as a biological weapon, which presume an infectious dose of 10 organisms, are negatively biased.”	[27]
		“The dose for 50 % infection (InfD50 %) in human subjects is 1.18 bacteria (95 % credible interval (CI) 0.76–40.2)…The median probability of infection from a single bacterium in humans is 0.44 (95 %CI 0.044–0.59)”	[29]
		“Using human study data conducted in the 1950's we conclude that the dose required for a 50 % probability of infection is about 15 organisms, and that one inhaled organism of *C. burnetti* (sic) can cause infection in 5 % of the exposed population.”	[30]

### 3.1 Infectious dose

The infectious dose required to cause *C. burnetii* infection in humans remains inconsistently defined, although it is well established that only a low number of organisms is necessary to initiate infection. When evaluated for bioweapons risk assessment, the effective infectious dose for *C. burnetii* was estimated to be 10 organisms [27]. A human challenge study in the early 1960s estimated infectious dose using relative measures—specifically, serial dilutions of infected egg slurry [28]. To approximate an absolute bacterial dose, data from this human study were compared with those from a concurrent guinea pig challenge using the same inoculum and exposure parameters. These were calibrated against a recent guinea pig aerosol challenge study employing quantified bacterial loads [29]. By aligning the dose–response relationships in guinea pigs across the two studies, researchers inferred the bacterial concentration in the original human exposures. Using these estimates, a hierarchical Bayesian framework was applied to jointly model the human dose–response relationship for airborne *C. burnetii* exposure. The median dose estimated to cause infection in 50 % of human subjects (ID₅₀) was 1.18 bacteria (95 % credible interval [CI]: 0.76–40.2), while the dose associated with illness in 50 % of cases was 5.58 bacteria (95 % CI: 0.89–89.0). The probability that a single viable bacterium would cause infection in a human was estimated at 0.44 (95 % CI: 0.044–0.59), with the probability of illness at 0.12 (95 % CI: 0.0006–0.55) [29]. In contrast, Heppell et al. (2017) [30] developed a human time-dose response model using a mechanistic birth-death-survival framework and fitting parameters to both historical and experimental data. Their model estimated a median inhaled ID₅₀ of 15 organisms (95 % CI: 11–21), with a retained dose of 1.86 organisms being sufficient to infect 50 % of individuals. They also concluded that a single inhaled bacterium has a 5 % chance of causing infection, aligning with in vitro and animal studies, which estimate that infectious doses can be as low as 1–10 organisms. Comparatively, humans exhibit greater susceptibility to *C. burnetii* than guinea pigs, with a lower median infectious dose (1.18 vs. 10.69 bacteria) and a higher probability of infection from a single organism (0.44 vs. 0.06) [29]. Nonetheless, the human dose–response curve is more variable, likely due to greater individual heterogeneity. In guinea pig models, intraperitoneal inoculation with a single bacterium leads to infection with a probability of approximately 0.9 [27].

### 3.2 Risk groups and biocontainment requirements

*C. burnetii* is classified as a risk group 3 (RG3) pathogen in most jurisdictions, including the USA (Centers for Disease Control and Prevention, 2020), Canada [31], Australia [32], and the European Union [33]. The US CDC Biosafety in Microbiological and Biomedical Laboratories 6th edition (BMBL6) [34] recommends using Biosafety Level 3 (BSL-3) practices and facilities for various procedures involving *C. burnetii*, although the biocontainment level depends on the activity. BSL-3 procedures and facilities are advised for activities such as inoculation, incubation, and harvesting of *C. burnetii*, necropsies of infected animals, and handling of infected tissues. Since infected rodents may shed the organisms through urine or faeces, animals infected experimentally should be maintained under ABSL-3 (Animal BSL-3) conditions. BSL-3 laboratories must be equipped with directional airflow, sealed workspaces, HEPA-filtered exhaust systems, and certified Class II biosafety cabinets to prevent aerosol exposure. Staff must undergo specialised training in BSL-3 protocols, including proper use of personal protective equipment (PPE), aerosol containment, waste decontamination, and emergency procedures (WHO, 2020). Importantly, due to the persistence of *C. burnetii* in the environment and its classification as a Category B bioterrorism agent, laboratory personnel should be made fully aware of the pathogen's epidemiology and risk profile. Regular competency assessments, incident reporting systems, and interdisciplinary training involving clinical and veterinary microbiology teams are essential to maintaining safety and ensuring diagnostic accuracy.

BSL-2 practices and facilities are recommended for non-propagative diagnostic laboratory procedures, including serological examinations, PCR, and staining of impression smears [34].

### 3.3 Select agent requirements and biothreat potential

In the United States, *C. burnetii* is classified as a Select Agent under the Code of Federal Regulations (42 CFR Part 73), which specifies the requirements for the possession, storage, use, and transfer of Select Agents and is considered a Category B biothreat agent [34]. However, a specific plaque-purified clonal isolate of an avirulent (phase II, Nine Mile Strain, plaque purified clone 4) strain is exempt from the Select Agent Regulations and may be safely handled under BSL-2 conditions.

### 3.4 Relative biorisk associated with sample type

Samples collected for the detection of *C. burnetii* can vary greatly in bacterial load and associated biosafety risks. Recognising these differences across sample types is essential for proper biosafety management and laboratory protocols. Routine clinical or environmental samples such as blood, serum, or dust often contain low to moderate levels of the pathogen and can usually be handled under BSL-2 conditions with standard precautions. In contrast, materials like placentas, birth fluids, and reproductive tissues from infected ruminants contain extremely high concentrations of *C. burnetii*—often exceeding 10^9^ organisms per gram—and pose a significant aerosol transmission risk [6,35]. Because of this heightened threat, such specimens require enhanced containment measures, including processing under BSL-3 conditions with strict engineering controls and personal protective equipment [34].

### 3.5 Large-scale Q fever outbreaks and lessons learned

One of the most significant Q fever outbreaks occurred in the Netherlands between 2007 and 2010, resulting in over 4000 confirmed human cases and at least 74 deaths, making it the largest recorded outbreak of *C. burnetii* infection globally [36]. The outbreak was primarily linked to intensive goat farming in densely populated areas, where infected birth products and aerosolised bacteria spread from farms to surrounding communities [37]. Critical shortcomings included delayed recognition of the zoonotic threat, poor coordination between veterinary and public health sectors, insufficient biosecurity on farms, and a lack of early mandatory notification systems [38]. Moreover, public health messaging and risk communication to residents were initially inadequate. These missteps delayed effective interventions such as culling, vaccination of livestock, and farm quarantines. The Dutch experience underscored the importance of One Health approaches, early warning systems, and clear biosafety protocols to prevent similar outbreaks in the future. It also highlighted the necessity of managing high-risk materials—like placental tissue—with heightened containment to prevent environmental dissemination and large-scale human exposure.

## 4 Prevention

A summary of biosafety and biosecurity requirements for the prevention of laboratory or occupationally acquired *C. burnetii* infections is presented in Table 3. The prevention of Q fever, particularly in laboratory and occupational settings, relies on a multifaceted approach integrating biosafety protocols, vaccination, PPE, and environmental control measures. BSL-2 containment measures are adequate for most routine diagnostic activities to mitigate the risk of laboratory-acquired infections. However, including in vitro culture in diagnostic procedures necessitates heightened awareness among laboratory personnel, as *C. burnetii* poses a significant biohazard. While initial isolation is permissible for diagnostic purposes within BSL-2 settings, all subsequent amplification or experimental manipulations must occur in BSL-3 facilities. BSL-3 procedures and biocontainment requirements for propagation activities ensure that, in the unlikely event of a significant infectious spill, aerosolised *C. burnetii* organisms remain contained within the laboratory. Additionally, any air released from the facility is HEPA-filtered to prevent contamination of the external environment.

**Table 3:** Summarised Biosafety and Biosecurity Requirements for Prevention of Laboratory or occupationally acquired *Coxiella burnetii* infections.

Category	Requirement	Details
Risk Group	Risk Group 3 (RG3)	Classified in most jurisdictions, including the USA, Canada, Australia, and the EU.
Biosafety Level (BSL)	BSL-3 (Propagation)	High-risk procedures - inoculation, cultivation, and harvesting of *C. burnetii*.
	BSL-2 (Diagnostics)	Non-propagative procedures - diagnostic activities, such as PCR or serology.
Animal Biosafety Level (ABSL)	ABSL-3	Handling infected animals due to shedding of the pathogen in faeces, urine, and other excretions.
Personal Protective Equipment (PPE)	Mandatory	Includes gloves, gowns, respiratory protection (e.g., N95 masks), and safety goggles or face shields.
Vaccination	Recommended	Pre-exposure vaccination (e.g., Q-VAX in Australia) for laboratory workers at risk.
Access Control	Restricted	Limited access to trained personnel, reinforced by electronic or physical security measures.
Disinfection	Chemical	70 % ethyl alcohol, 5 % chloroform, and 10 % bleach (with ≥30 min exposure).
	Gaseous	Paraformaldehyde gas at 0.3 g/ft^3^ with 80 % relative humidity for overnight decontamination.
Decontamination of Waste	Autoclaving or Incineration	Preferred methods for infectious materials.
	Chemical Soaking	For facilities without autoclaves: soak in 10 % bleach or 95 % ethanol for at least 24 h.
Aerosol Containment	HEPA filtration	Use of HEPA-filtered biosafety cabinets (Class II or III) for all manipulations involving aerosols.
Biothreat Classification	Category B Select Agent	Subject to regulations under 42 CFR Part 73 in the USA.
Training and Competency	Mandatory	Regular training and competency testing in handling RG3 pathogens and use of PPE.
Incident Reporting	Immediate Reporting	Notify biosafety officers and public health authorities for exposure incidents or breaches.
Environmental Controls	Ventilation	Directional airflow in laboratories, maintaining negative pressure to prevent aerosolized pathogen escape.
Storage and Transport	Secure Storage	Use of locked freezers or facilities for pathogen storage, with access restricted to authorized personnel.
	Secure Transport	Packaging compliant with international regulations (e.g., UN 2814 for Category A infectious substances).

### 4.1 Vaccination in humans

Vaccination is a highly effective and crucial strategy in preventing Q fever outbreaks. Q-VAX is currently the only commercially available vaccine for Q fever and has obtained licensure only within Australia [39]; a whole cell preparation derived from the formalin-inactivated phase 1 Henzerling strain, manufactured by CSL Seqirus in Australia [40]. The vaccine must be administered at least two weeks prior to exposure to provide effective protection. The vaccine elicits a durable immune response after vaccination [41]. Both a serological test and an intradermal skin test (using diluted vaccine) must be performed on the vaccinee before vaccination [40]. To detect the likelihood of adverse reactions in persons having had prior contact with the bacterium, both the antibody and skin tests must be negative before vaccination.

### 4.2 Personal protective equipment

A strong correlation has been observed between the reduced incidence of *C. burnetii* infections and adherence to appropriate PPE protocols. Studies involving veterinarians, laboratory personnel, and agricultural workers confirm that consistent use of PPE significantly mitigates the risk of infection [[42], [43], [44], [45]]. Essential PPE items, such as gloves, N95 respirators, safety goggles, and gowns, have proven highly effective in minimising transmission risks in laboratory and field settings.

### 4.3 Disinfection and decontamination

A summary of disinfectants that are effective for *C. burnetii* decontamination is presented in Table 4. Limited information is available regarding the efficacy of chemical disinfectants for the inactivation of *C. burnetii*. *C. burnetii* was effectively inactivated within 30 min using either a 70 % concentration of ethyl alcohol, 5 % chloroform, or 5 % Enviro-Chem (see Table 4 active ingredients) [46]. Guidance from the American Society for Microbiology for minor *C. burnetii* spills is to cover with absorbent paper, then flood with 70–95 % alcohol or 5 % MicroChem-Plus (see Table 4 for active ingredients) and allow 30 min before cleanup [47]. The same guidance recommends that *C. burnetii* clinical material be autoclaved or incinerated on-site, and if no autoclave is available, contaminated items should be soaked in 95 % alcohol, 10 % bleach, or 10 % formalin for 24 h [47]. Gaseous disinfection using paraformaldehyde polymer gas at 80 % relative humidity and 0.3 g/ft^3^ was used to inactivate *C. burnetii* (10^8^) in a confined space (88 cubic feet). Radiation has proven effective with doses between 0.64 and 1.2 kilograys (kGy) that achieved a 90 % reduction of *C. burnetii* at a temperature of −79 °C [48].

**Table 4:** Detailed disinfection and inactivation methodologies for *Coxiella burnetii.*

Method	Evidence (direct quote)	Reference
Chemical	“Overnight exposure to humidified (80 % relative humidity) formaldehyde gas, generated by depolymerizing paraformaldehyde at a rate of 0.3 g/ft3 of space within a sealed 88-ft3 chamber, effectively inactivated log8 *C. burnetii* on the surface of membrane filters”	[46]
	“By contrast, similar concentrations of *C. burnetii* were completely inactivated within 30 min by either 70 % ethyl alcohol, 5 % chloroform, or 5 % Enviro-Chem” (Note: Enviro-Chem comprises Ethanol, Alkyl Dimethyl Benzyl Ammonium Chloride, Didecyl Dimethyl Ammonium Chloride, Dimethyl Octyl Amine Oxide)”	[46]
	“Minor spills should be covered with absorbent paper, such as paper towels, and then flooded with 70–95 % alcohol or 5 % MicroChem-Plus (a dual quaternary ammonium compound, pH 10.9), which should be allowed to act for 30 min before cleanup”	[47]
	“Specimens containing the organism should be autoclaved, incinerated on-site or submitted to the designated LRN Reference Laboratory for disposal. If no autoclave is available, contaminated items should be soaked in 95 % ethanol, 10 % bleach, or 10 % formalin for 24 h.	[47]
Radiation	“The gamma radiation inactivation kinetics for *Coxiella burnetii* at −79 degrees C were exponential. The radiation dose needed to reduce the number of infective *C. burnetii* by 90 % varied from 0.64 to 1.2 kGy depending on the phase of the micro-organism, purity of the culture and composition of suspending menstruum.”	[48]
Filtration	“Filtration using a 0.1 μm filter was effective: Both the F and the FH sample showed an immediate increase in Cp-value (approximately 5 cycles) compared to the untreated sample, and were negative in PCR from day 7 (F) and 10 (FH) onwards, i.e. no growth was observed.”	[57]
Pasteurisation of milk	“…the temperature of 145 °F. for 30 min will eliminate the organism. The pasteurisation of milk according to the present standards for HTST equipment of 161 °F. for 15 s seems adequate to destroy *C. burnetii*.”	[58]
	“After heat treatment of the field isolates, no viable bacteria were detectable at temperatures between 68 and 75 °C, according to the CFU assay.”	[59]

## 5 Discussion

This scoping review underscores the complexity and critical importance of managing biosafety risks associated with *C. burnetii*, particularly in laboratory and occupational settings. *D*ue to its environmental resilience, low infectious dose, and diverse transmission routes, *C. burnetii* presents unique challenges. Despite significant advances in understanding its characteristics and implementing biosafety measures, critical gaps in evidence and practice remain, especially in resource-constrained settings.

### 5.1 Laboratory biosafety practices: achievements and challenges

The findings affirm the crucial importance of risk-based biosafety practices, as highlighted in the WHO LBM4 and CDC's BMBL6. These frameworks provide a foundation for the safe laboratory handling of *C. burnetii* and recommend BSL-3 practices for propagative work while suggesting BSL-2 for diagnostic activities [34]. Many laboratories—especially in low- and middle-income countries (LMICs)—face significant challenges in achieving these standards. Establishing BSL-3 laboratories demands substantial financial investment for construction, maintenance, and equipment such as HEPA filtration, negative pressure systems, and biosafety cabinets. Moreover, supporting BSL-3 operations requires reliable infrastructure (e.g., continuous power supply, HVAC systems) and a skilled workforce trained in advanced biosafety protocols—both often scarce in resource-limited settings [1]. Consequently, laboratories in such environments must adopt risk-based mitigation strategies. These include strict adherence to enhanced BSL-2 procedures—such as using sealed centrifuge cups, respiratory protection, controlled access, and rigorous decontamination protocols—when handling potentially high-risk samples like animal birth products or tissues. Developing SOPs that suit available resources, alongside external quality assurance programmes and regional training initiatives, can further minimise occupational exposure and diagnostic errors. Ultimately, flexible and scalable biosafety strategies are vital to closing the gap between international guidelines and local laboratory capabilities.

### 5.2 Laboratory-acquired infections: improvements or underreporting?

Between 1910 and 1950, *C. burnetii* ranked as the second most commonly reported LAI globally, with 96 documented cases occurring in 65 laboratories across 18 countries [12]. This historical data underscores the pathogen's high infectivity and potential for aerosol transmission in laboratory settings. While no laboratory-acquired cases were officially reported between 2001 and 2021, this absence is more likely due to underreporting and limited surveillance infrastructure than the elimination of risk, particularly in regions with inadequate biosafety protocols or limited diagnostic capacity [15]. The continued use of serological and molecular diagnostics in BSL-2 environments for non-propagative work reinforces the importance of risk awareness and biosafety vigilance even in the absence of reported incidents.

### 5.3 Vaccination as a preventive tool: limitations and future directions

Vaccination remains a cornerstone strategy for preventing Q fever, particularly among high-risk groups such as veterinarians, slaughterhouse workers, and laboratory personnel working with *C. burnetii*. The Q-VAX vaccine, derived from the formalin-inactivated *C. burnetii* Henzerling strain, has demonstrated efficacy in preventing infection and is currently the only licensed vaccine for human use, available exclusively in Australia [40]. However, pre-vaccination screening is required to avoid adverse reactions in individuals previously exposed to the pathogen, complicating its broader adoption. Expanding global access to Q-VAX or developing alternative immunisation strategies is essential to protect at-risk populations. A vaccine that does not require the vaccinee to be pre-tested would be a significant advance. Additionally, it's worth noting that substantial global efforts are underway to develop a next-generation Q fever vaccine that could achieve worldwide licensure. This includes research conducted by the Australian Rickettsial Reference Laboratory, which aims to address current vaccine limitations and enhance protective efficacy, safety, and accessibility.

### 5.4 Environmental and occupational risks: persistent challenges

The resilience of *C. burnetii* under varied environmental conditions adds complexity to its management. The bacterium's ability to persist in aerosols, milk, and contaminated clothing highlights the multifaceted nature of occupational and environmental risks [23,49]. For instance, outbreaks have been linked to aerosol transmission from infected animals or their environments, with specific incidents arising from air conditioning systems and livestock exposure [16,50]. Preventative measures such as pasteurisation, PPE, and robust decontamination protocols have demonstrated effectiveness, yet their consistent application remains challenging. These findings highlight the need to adapt mitigation strategies to local environmental conditions.

### 5.5 Evidence gaps and research priorities

This review identifies critical evidence gaps, particularly in the efficacy of specific disinfection methods and the contributions of environmental variables to pathogen spread. The limited comparative data on chemical, thermal, and radiation-based disinfection methods for *C. burnetii* in diverse settings warrant further investigation [46,48]. Additionally, the underreporting of LAIs and challenges in vaccine accessibility point to the need for enhanced surveillance and global collaboration. Research priorities include:

1. Enhanced Surveillance: Establishing robust reporting systems to accurately assess LAI prevalence and inform interventions.
2. Disinfection Efficacy: Investigating optimal methods for pathogen inactivation across laboratory and environmental contexts.
3. One Health Integration: Developing interdisciplinary approaches integrating human, animal, and environmental health.

## 6 Conclusion

*C. burnetii* remains a challenging pathogen in laboratory research due to its low infectious dose and resilience. This manuscript underscores the need for rigorous application of risk-based biosafety protocols tailored to specific laboratory activities. By integrating advanced disinfection methods, scalable infrastructure, and vaccination strategies, laboratories can significantly reduce the risks of laboratory-acquired infections. Future efforts should focus on addressing gaps in disinfection efficacy data, enhancing vaccine accessibility, and ensuring global compliance with biosafety standards. Strengthening training programs and fostering international collaboration will be pivotal to achieving safer research environments for *C. burnetii*.

## CRediT authorship contribution statement

**S.D. Blacksell:** Writing – review & editing, Writing – original draft, Supervision, Project administration, Methodology, Funding acquisition, Formal analysis, Conceptualization. **K.K. Le:** Writing – review & editing, Writing – original draft, Data curation. **L.J. Gleeson:** Writing – review & editing. **J. Stenos:** Writing – review & editing. **S.R. Graves:** Writing – review & editing. **N.P.J. Day:** Writing – review & editing, Funding acquisition.

## Funding

This research was funded in part by the 10.13039/100010269Wellcome Trust [Grant number 220211/Z/20/Z]. For the purpose of open access, the author has applied a CC BY public copyright license to any Author Accepted Manuscript version arising from this submission.

## Declaration of competing interest

The authors declare that the research was conducted without any commercial or financial relationships that could be construed as a potential conflict of interest.

## Data Availability

There is no specific dataset related to this study.
